# Optogenetically Controlled Activity Pattern Determines Survival Rate of Developing Neocortical Neurons

**DOI:** 10.3390/ijms22126575

**Published:** 2021-06-19

**Authors:** I. Emeline Wong Fong Sang, Jonas Schroer, Lisa Halbhuber, Davide Warm, Jenq-Wei Yang, Heiko J. Luhmann, Werner Kilb, Anne Sinning

**Affiliations:** Institute of Physiology, University Medical Center Mainz, Johannes Gutenberg University, Duesbergweg 6, 55128 Mainz, Germany; emma.emelinewong@gmail.com (I.E.W.F.S.); jonas.schroer@uni-mainz.de (J.S.); lhalbhub@uni-mainz.de (L.H.); daviwarm@uni-mainz.de (D.W.); yangj@uni-mainz.de (J.-W.Y.); luhmann@uni-mainz.de (H.J.L.); wkilb@uni-mainz.de (W.K.)

**Keywords:** cortex, development, apoptosis, optogenetics, activity pattern, burst, Bax, mouse

## Abstract

A substantial proportion of neurons undergoes programmed cell death (apoptosis) during early development. This process is attenuated by increased levels of neuronal activity and enhanced by suppression of activity. To uncover whether the mere level of activity or also the temporal structure of electrical activity affects neuronal death rates, we optogenetically controlled spontaneous activity of synaptically-isolated neurons in developing cortical cultures. Our results demonstrate that action potential firing of primary cortical neurons promotes neuronal survival throughout development. Chronic patterned optogenetic stimulation allowed to effectively modulate the firing pattern of single neurons in the absence of synaptic inputs while maintaining stable overall activity levels. Replacing the burst firing pattern with a non-physiological, single pulse pattern significantly increased cell death rates as compared to physiological burst stimulation. Furthermore, physiological burst stimulation led to an elevated peak in intracellular calcium and an increase in the expression level of classical activity-dependent targets but also decreased Bax/BCL-2 expression ratio and reduced caspase 3/7 activity. In summary, these results demonstrate at the single-cell level that the temporal pattern of action potentials is critical for neuronal survival versus cell death fate during cortical development, besides the pro-survival effect of action potential firing per se.

## 1. Introduction

During early development, neurons are produced in excess, and neuronal population sizes are only later reduced by apoptotic cell death. This fundamental process is important for the maturation of functional neuronal networks and thus has to be tightly regulated [1,2,3]. While genetic programs, trophic support, and pro-/anti-apoptotic factors contribute to the regulation of neuronal apoptosis [4,5], the influence of neuronal activity in the regulation of apoptosis gathers particular interest [6,7,8]. Previous studies using pharmacological manipulations demonstrated that neuronal activity has a major impact on cell death and survival rates in developing cortical networks [9,10,11]. Whereas blockade of neuronal activity strongly increased neuronal death rates [9,10,12], elevated neuronal activity was generally associated with a decrease in neuronal death [13,14,15]. The importance of individual activity states at the cellular level for this process was supported by in vivo imaging experiments in the cortex and in vitro data from hippocampal neurons showing that inactive neurons are more likely to die than active neurons [16,17]. Recently it became evident that neuronal activity is of special importance for the regulation of cell death rates in distinct neuronal cell types [17,18,19,20,21]. In particular, it serves to eliminate transient neuronal populations during early cortical development [18,19,20,22], to match the ratio of cortical interneurons and pyramidal cells [17], and to define neuronal population sizes across different cortical areas [23]. Thus, a better understanding of the activity-dependent neuronal apoptosis, its regulatory mechanisms, as well as its importance for brain development is emerging (for review, see [3,6,8]). However, many critical questions in this fundamental developmental process remain open. Population-based approaches with pharmacological or electrical stimulation suggest correlations of distinct neuronal activity patterns with respective apoptotic rates [13,23,24]. However, from these experiments, the contribution of intrinsic electrical activity states of individual neurons remains indistinguishable from the role of its network-dependent synaptic inputs.

The aim of the present study was, therefore, to investigate the potential pro-survival effect of specific electrical activity patterns and to study if the temporal distribution of action potentials is critical for mediating the survival of developing cortical neurons. For this purpose, the spontaneous network-driven activity of primary cortical cultures was, upon blockade of glutamatergic and GABAergic synaptic inputs, optogenetically replaced by chronic LED-based light stimulation using two different stimulus paradigms (physiological burst vs. non-physiological regular single-pulse stimulation) with an identical number of stimuli. Numerous previous studies have shown that spontaneous network bursts (e.g., spindle and gamma bursts) are the physiological activity pattern dominating during early cortical development [25,26,27,28].

The effects of this persistent optogenetic stimulation, combined with electrical recordings of the resulting activity, were quantified by cell fate assessment over the time course of three days, using time-lapse imaging of neurons expressing a nuclear H2B-coupled mCherry signal, as well as gene expression analysis, calcium imaging, and monitoring of caspase activity. The results of these experiments suggest that action potential firing with a physiological burst pattern is neuroprotective at the single neuron level, while identical activity levels provided with un-physiological temporal patterns are less effective.

## 2. Results

### 2.1. Action Potential Firing of Cortical Neurons Promotes Neuronal Survival throughout Development

**Figure 1:**
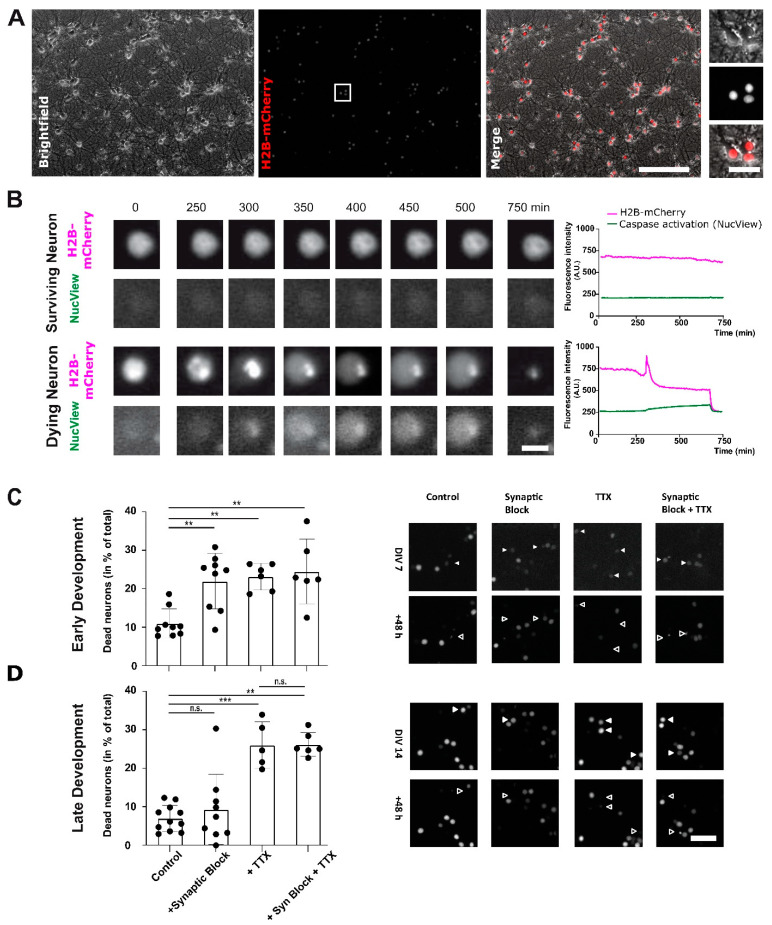
Live cell imaging of H2B-mCherry labeled primary cortical neurons can be used to quantify neuronal survival throughout development and upon pharmacological modulation of neuronal activity. (**A**) Representative primary cortical culture with nuclear fluorescence labeling using a transgenic mCherry-tagged histone cluster protein (H2B-mCherry) at DIV16. (**B**) Nuclear H2B-mCherry signal (magenta) enables the identification of hallmarks of apoptosis: chromatin condensation and DNA fragmentation. Longitudinal imaging of H2B-mCherry positive nuclei in cortical cultures shows constant signal intensity and appearance in surviving neurons without a relevant change in NucView signal (green). In contrast, dying neurons can be clearly identified by a change in H2B-mCherry signal appearance (nuclear fragmentation, swelling and condensation) followed by a strong decline in signal intensity or its disappearance. (**C**) Survival rates in primary cortical neurons in the early developmental period (DIV7-9) are significantly diminished upon pharmacological blockade of synaptic transmission (10 µM CNQX, 50 µM d-APV, 10 µM Gbz; *n* = 9/9; *p* < 0.01), of action potential generation (1 µM TTX; *n* = 6; *p* < 0.01) or after blockade of both processes (*n* = 6; *p* < 0.01). In the representative images, full arrowheads point to healthy H2B-mCherry positive nuclei at the start of the experiment and the unfilled arrowheads mark its former location after cells died. (**D**) In the late developmental period (DIV14-16), neuronal survival rates are no longer diminished by the blockade of synaptic transmission (*n* = 11/11; *p* < 0.05), but still, more neurons die during the blockade of neuronal firing (*n* = 9; *p* < 0.001). Scale bars represent 100 µm (**A**,**C**,**D**), 20 µm (insert (**A**)), and 10 µm (**B**). Data points indicate the mean ± SEM. **, *** indicate *p*-values of 0.01, and 0.001, respectively.

Cell death or survival can be monitored and quantified by many different cell biological methods, e.g., by quantitative analysis of colorimetric viability or metabolic assay, by visualizing alterations in membrane composition, by detecting DNA fragmentation, by identifying caspase-3 activation, or by mere counting of surviving neurons at the endpoint [29]. These methods mostly detect transient cellular events (e.g., cleavage of Casp-3) or only allow an endpoint analysis (e.g., TUNEL assay). This implicates the disadvantage that small but physiologically relevant differences in cell survival/death rates can be overseen due to the transient nature of analyzed parameters or to large variabilities in the initial states of individual experiments. In contrast, chronic or repeated imaging of neurons allows real-time monitoring of cell survival and death as well as the temporal aspects of individual cell fates. For this purpose, we overexpressed a fluorophore-tagged Histone cluster 1 protein (H2B-mCherry) in primary cortical neurons to clearly identify individual cells (Figure 1A), but also to trace longitudinal changes in nuclear morphology and chromatin condensation (Figure 1B). Both events are hallmarks of apoptosis [30].

To test whether dying and dead neurons can be clearly identified in H2B-mCherry-transduced cortical cultures, we performed repeated time-lapse imaging of the fluorescent neurons that express this transgenic fusion protein. Neurons undergoing spontaneous cell death during the time course of the experiment displayed a clear change in chromatin organization as revealed by an altered H2B-mCherry signal distribution in the nucleus followed by a prominent loss in the appearance and intensity of the signal. In parallel to the changes in nuclear morphology (H2B-mCherry signal) and before the final disappearance of the fluorescent-tagged nuclei, neurons showed strong activation of Caspase-3—a key player of apoptosis—as indicated by a transient increase in NucView488 signal (Figure 1B). In contrast, surviving neurons presented a stable and homogenous H2B-mCherry signal in its nucleus and were negative for NucView488 signal throughout the 72 h recording interval. Thus, neuronal apoptosis resulted in a prompt and obvious change of the histone-tagged fluorescent signal and finally in the disappearance of nuclear profiles. To confirm that a quantification of the H2B-mCherry signal can also be used to determine neuronal cell death rates, neurons were treated with staurosporine, a well-known inducer of apoptosis, and cell survival was quantified by repeated imaging of the neuronal cultures. Neurons under control conditions showed a slight, developmental increase in H2B-mCherry signal intensity (normalized nuclear signal intensity 0 h 1.00 ± 0.04; 24 h 1.04 ± 0.05; 48 h 1.18 ± 0.07; 72 h 1.29 ± 0.08; *n* = 30 cells; One-Way ANOVA *p* = 0.0023). In contrast, neurons treated with 1.5 µM staurosporine showed a significant decrease in signal intensity already after 24 h (0 h 1.00 ± 0.05; 24 h 0.68 ± 0.04; 48 h 0.058 ± 0.02; 72 h 0.59 ± 0.01; *n* = 30 cells; *p* < 0.0001 One-Way ANOVA) demonstrating the induction of neuronal cell death in the staurosporine-treated cultures. When not only signal intensity but also morphological changes of the nucleus were considered, about 60% of nuclei in staurosporine-treated neurons showed clear morphological hallmarks of apoptosis (20% nuclear fragmentation, 23% pyknosis, and for 20% only minor remnants of the nucleus) already after 6 h. After 24 h, 100% of neurons were classified as apoptotic or dead based on signal intensity and signal appearance (10% nuclear fragmentation, 40% pyknosis, and for 50% only minor remnants of the nucleus). In contrast, nuclei of neurons in untreated control cultures did not show typical hallmarks of apoptosis or death after 6 h. After 24 h, only a few cells showed either nuclear fragmentation (3.33%) or pyknosis (6.66%). We conclude from these results that tracking of H2B-mCherry positive nuclei can be used to visualize cell survival/death of individual neurons in real-time but also allows the quantification and comparison of cell death rates across experimental groups.

Using the quantification of H2B–mCherry positive neurons, we next investigated the impact of neuronal activity on the survival of primary cultured neurons during early and late development in vitro. In line with previous in vivo data [17,23,31], the relative proportion of dying neurons in vitro was significantly higher in early development at DIV7-9 (10.99 ± 1.28%, *n* = 9 fields of view) compared to later development at DIV14-16 (7.15 ± 1.04%; *n* = 11 fields of view, *p* = 0.03 students t-test). In the early development period (DIV7-9), neuronal survival was significantly impaired if GABAergic and glutamatergic synaptic inputs were pharmacologically blocked by the combined application of 10 µM Gabazine, 10 µM CNQX and 50 µM d-APV, respectively (Figure 1C). The fraction of dying neurons was significantly (*p* = 0.0004 One-Way ANOVA; *p* < 0.01 posthoc test) increased from 10.99 ± 1.28% under control conditions to 22.13 ± 2.44% (*n* = 9 fields of view from 3 cultures each) upon this synaptic blockade. Blockade of action potentials with 1 µM TTX had a comparable effect as it significantly (*p* < 0.01 posthoc test) increased the fraction of dying neurons to 23.23 ± 1.43% (*n* = 6 fields of view). The combined blockade of action potential firing and synaptic inputs demonstrated that these effects were non-additive (24.71 ± 3.49%; *n* = 6 fields of view; *p* < 0.01 posthoc test). These observations confirm that in the early developmental period neuronal survival critically depends on neuronal firing as well as synaptic inputs from the surrounding neuronal network. At a later developmental time point (DIV14-16; Figure 1D), survival of cortical neurons in vitro was still impaired if neuronal firing is blocked by 1 µM TTX. Under this condition the fraction of dying neurons was significantly (*p* < 0.0001 One-Way ANOVA; *p* < 0.001 posthoc test) increased to 26.45 ± 2.72% (*n* = 9 fields of view) from the control value of 7.15 ± 1.04% (*n* = 11 fields of view). However, at DIV14-16, neuronal survival rates of primary neurons were no longer significantly diminished in the absence of synaptic inputs (9.47 ± 3.08%; *n* = 11 fields of view; *p* > 0.05 posthoc test). The combined treatment of synaptic blockers and TTX had a comparable effect as TTX alone (17.97 ± 5.11%; *n* = 6 fields of view; *p* > 0.05 posthoc test).

In parallel to the finding that at the later developmental period, synaptic blockade did not affect neuronal survival rates, MEA-based electrical recordings were performed. These recordings showed that primary cortical cultures under persistent blockade of glutamatergic and GABAergic receptors at the late developmental phase surprisingly still showed spontaneous neuronal activity. Multi-unit activity was only reduced significantly after at least 48 h of combined application of glutamatergic and GABAergic receptor blockers (1 h control 125.73 ± 33.85%, blocked 88.39 ± 42.11%; 24 h control 88.42 ± 16.90% blocked 47.44 ± 16.08%; 48 h control 153.14 ± 14.67% blocked 27.13 ± 11.80%; *n* = minimum of 3 cultures per group; *p* = 0.81 (1 h), *p* = 0.20 (24 h) and *p* = 0.004 (48 h) students *t*-test). This unexpected stability of neuronal network activity could be explained by the oppositional effects of blockade of inhibitory and excitatory transmission [32] and also by the fact that, especially during this phase of development, synaptic neuronal transmission was strongly supported by electrical synapses [33,34].

Taken together, these data demonstrate that action potential firing is necessary for neuronal survival during early and late development, whereas the mere blockade of synaptic inputs does not significantly affect neuronal survival at DIV14-16.

### 2.2. In Late Development Action Potential Firing of Cortical Neurons Is Dominated by Bursting Pattern

**Figure 2:**
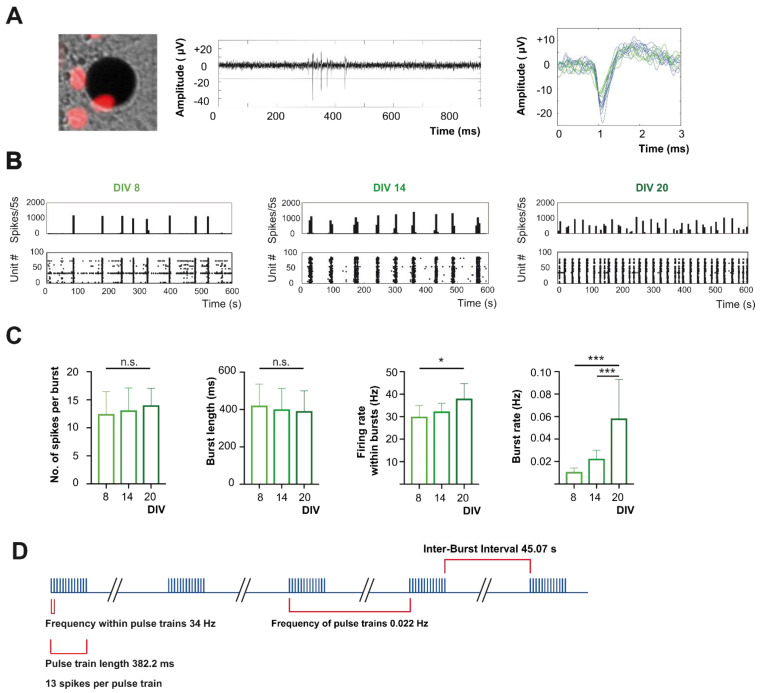
Design of stimulation protocol based on characteristics of spontaneous network activity during late development. (**A**) H2B-mCherry positive neurons on one of the 120 MEA-electrodes with the corresponding raw trace of electrical activity recorded. In this example, the electrode picks up action potentials with two distinct shapes above the detection threshold. Spike sorting algorithms grouped action potentials into two clusters and thus assigned them to two different putative neurons. (**B**) Example time histograms and raster plots of spontaneous activity at DIV8, DIV14, and DIV20. Time histograms spike rates were calculated in 5 s time bins. Note that during late development, neuronal firing patterns of cortical cultures are dominated by coherent bursting activity. (**C**) Quantification of properties of neuronal activity patterns. The number of spikes per burst and the burst length appeared to be stable between DIV8 and DIV20 (*n* = 20/5/5; *p* > 0.05). The firing rate in bursts increases significantly (*p* = 0.012) between DIV8 and DIV20. The firing rate of bursts increases significantly between DIV8 and DIV20 (*p* < 0.0001) and between DIV14 and DIV20 (*p* < 0.001). (**D**) Design of burst stimulation protocol mimicking spontaneous physiological activity. We based the burst stimulation protocol on the averages of the bursting parameters analyzed at DIV14. Data points indicate mean ± SEM and *, *** indicate *p*-values <0.05, and <0.001, respectively.

Developing cortical networks show a rich repertoire of activity patterns, which in vivo and in vitro share the appearance of correlated bursting activity as a fundamental feature [35,36]. To confirm that AAV-based transduction for the expression of the H2B-mCherry and Channelrhodopsin constructs did not affect the development of such correlated neuronal activity, we first analyzed the activity of these cultures from DIV6-20. The systematic observation that spikes could be recorded from electrodes that had only transduced neurons in their close vicinity (<50 µm, [37]) indicated that transgenic neurons could still fire action potentials (Figure 2A). Like in naïve cultures, the network activity of AAV-transduced cultures in the early developmental period (DIV6-9) was characterized by single spikes, which were partly synchronous. Within a few days, neuronal activity was dominated by strongly synchronized bursting activity (DIV11-15). This synchronized bursting activity state persisted as the cultures matured (DIV22, Figure 2B). Since the aim of the present study was to test whether the temporal patterning of spontaneous electrical activity contributes to the physiological maturation of neuronal networks, we decided to base the design of our stimulation protocols on characteristics of spontaneous activity. For this reason, we quantified the main features of correlated bursting activity in our culture at three developmental stages (DIV8, 14, and 20).

The burst rate (DIV8 0.01 ± 0.001 Hz, DIV14 0.02 ± 0.003 Hz, DIV20 0.06 ± 0.016 Hz, *n* = 20, 5 and 5 cultures; one-way ANOVA *p* < 0.05) increased significantly between DIV8 and DIV20 (*p* <0.0001 posthoc test) and between DIV14 and DIV20 (*p* = 0.001 posthoc test). The number of spikes per burst (DIV8 12.48 ± 0.89, DIV14 13.13 ± 1.78 and DIV20 14.05 ± 1.34) and the burst length (DIV8 421.3 ± 25.65, DIV14 401.3 ± 50.11 and DIV20 391.9 ± 48.33 ms) appeared to be stable within this developmental period (*p* > 0.05, one-way ANOVA respectively). However, the firing rate in bursts increased significantly between DIV8 and DIV20 (DIV8 30 ± 1.12 Hz, DIV14 32.36 ± 1.62 Hz, and DIV20 38.05 ± 2.3 Hz; *p* = 0.01 posthoc test). Based on the results of the analysis of spontaneous activity recorded at DIV14, we designed a burst stimulation protocol mimicking the physiological burst pattern of neuronal activity in this later developmental period. The burst stimulation protocol had the following parameters: (i) within a pulse train 13 light pulses were applied, based on an average of 13 spikes recorded within a detected burst. (ii) A stimulus frequency of 34 Hz, comparable to the mean firing frequency within recorded bursts of 32 Hz. This corresponds to an inter-stimulus interval within the stimulus trains of ca. 29.4 ms, which was close to the recorded mean inter-spike interval of 33 ms. (iii) A pulse train length of 338.2 ms, which resembled the recorded mean burst length of 400 ms. And (iv) a burst rate of 0.022 Hz, which recapitulated the measured mean burst rate of 0.022 Hz (Figure 2D). To determine whether the burst-like temporal structure of neuronal activity is critical for the regulation of apoptotic rates, we additionally designed a regular pulse pattern from these parameters. For this purpose, we redistributed 1030 light pulses within a 60 min-period evenly in time, resulting in a regular interstimulus interval of 3.496 s (corresponding to 0.286 Hz).

### 2.3. Optogenetic Light Stimulation Allows Physiological and Non-Physiological Stimulus Paradigms

**Figure 3:**
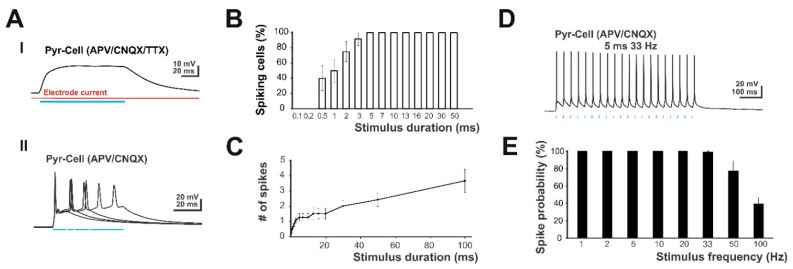
Patch clamp analysis of light-induced membrane responses in genetically modified cortical neurons expressing ChR2 and H2B-mCherry. (**A**) Patch clamp analysis of light-induced membrane responses in ChR2 transfected neurons. Panel I. Voltage-trace of a ChR2 transfected neuron upon 100 ms long illumination with blue LED light after inhibition of action-potential generation and synaptic transmission. The red trace illustrates the current of an unattached patch clamp pipette at a holding potential of 0 mV. Note that illumination induced a stable depolarization and had virtually no effect on the electrode alone. Panel II: Superimposed voltage traces of a ChR2 transfected neuron upon illumination for 10, 15, 20, and 40 ms after inhibition of synaptic transmission only. Note that illumination reliable induces action potentials and that at durations ≥ 15 ms, more than one AP was induced. (**B**) Response probability of the neuron upon stimulation using different stimulus durations. Note that reliable suprathreshold stimulations were induced at durations ≥ 5 ms. (**C**) Statistical analysis of the dependency between duration of illumination and the number of spikes. Note that between 5 and 15 ms duration, mainly a single AP was induced. (**D**) Voltage trace of a ChR2-positive neuron that was repetitively stimulated by light pulses. Note that this neuron reliably follows this individual burst stimulation. (**E**) Statistical analysis of spike probability at different stimulus frequencies. Note that AP generation reliably follows the repeated stimulation at frequencies of up to 33 Hz.

To confirm that AAV-based transduction resulted in a functional expression of ChR2 and to optimize the optogenetic stimulus parameters, we first performed single-cell patch clamp recordings on optically identified ChR2-positive neurons in cortical cultures at DIV14. Under pharmacological inhibition of action-potential generation and glutamatergic synaptic transmission (1 µM TTX; 10 µM CNQX and 60 µM d-APV), a 100 ms LED-based blue light illumination induced a stable depolarization in visually identified ChR2-positive neurons (Figure 3A upper panel). Control experiments revealed that the photostimulation with the used illumination parameters had virtually no effect on the electrode alone. The next experiments were performed to optimize the length of the photostimulation. In the presence of glutamatergic synaptic blockers (10 µM CNQX and 60 µM d-APV), illumination of ChR2- expressing neurons for 5 ms was sufficient to induce an action potential in all 9 recorded neurons (Figure 3A lower panel and Figure 3B). Up to a stimulus duration of 15 ms mainly single action potentials were evoked by the stimulation, while longer stimulation evoked multiple action potentials (Figure 3C).

In addition, we tested the voltage-response of neurons upon repeated light stimulation (5 or 10 ms stimulus length) with varying inter-stimulus intervals (Figure 3D,E). Cortical neurons at DIV14-16 could reliably follow burst stimulation with a frequency of up to 33 Hz (Figure 3E). If the stimulus frequency exceeded 33 Hz, spike probability decreased to 77.1 ± 11.6% (*n* = 7, *p* = 0.098) at 50 Hz and to 39.4 ± 7% (*n* = 7, *p* = 0.001) at 100 Hz. Thus, single-cell patch clamp recordings suggested that LED-based photostimulation with a stimulus duration of 5–10 ms allowed to reliably stimulate ChR2-positive neurons with a stimulus frequency of up to 33 Hz. Under this condition, the number of action potentials corresponded directly to the number of stimuli.

**Figure 4:**
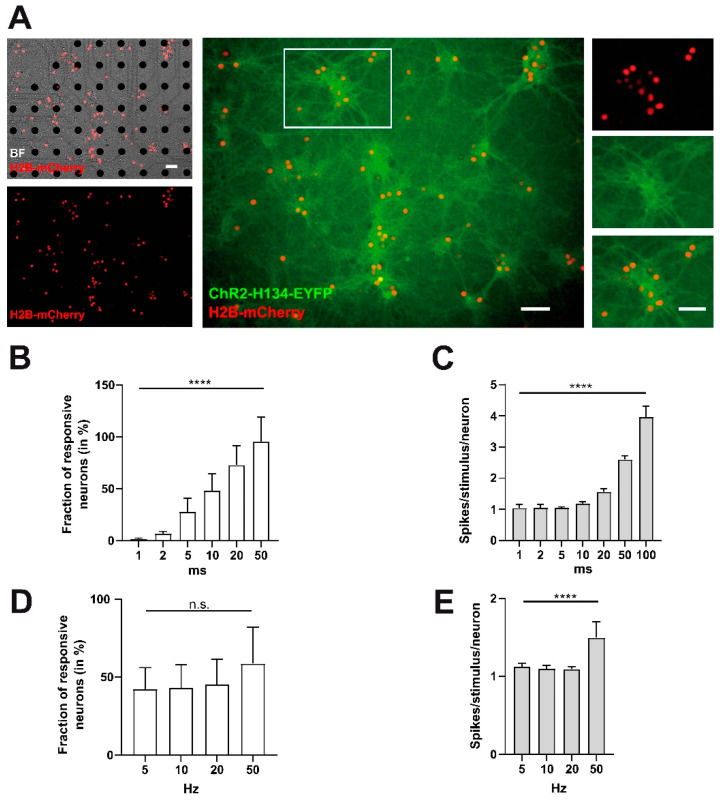
LED-based illumination of genetically modified cortical neurons on MEA allows optogenetic stimulation with physiological stimulus parameters. (**A**) Example culture of ChR2- and H2BmCherry positive neurons at the start of recording and stimulation (DIV14). Note that most neurons are co-transduced with both transgenes. (**B**) Effect of the pulse duration of optogenetic stimulation on extracellular responses of cortical cultures recorded by MEAs. The relative fraction of responding neurons increased with increasing pulse duration (*n* = 7 cultures; *p* < 0.0001). (**C**) In the responding neurons, mainly a single action potential was induced at stimulus durations ≤10 ms (*p* < 0.00001). (**D**,**E**) Effect of stimulus frequency using a pulse duration of 10 ms on the relative fraction of responding neurons (**D**) and the number of spikes per responding neurons (**E**). Note, neurons can follow the test stimuli with a frequency of up to 50 Hz. Scale bars represent 50 µm. Data points indicate the mean ± SEM and **** indicates *p*-value of <0.0001, respectively.

Next, we addressed the response efficiency parameters of primary neurons overexpressing ChR2 and H2B-mCherry on MEAs to LED-based light stimulation (Figure 4, *n* = 7 cultures). Extracellular MEA recordings showed that the fraction of neurons responding to the stimulation with 10 and 20 ms long light pulses was around 50% of the total number of units recorded before the synaptic blockade. Shorter stimuli (1–5 ms) resulted in less than 100% of expected responses and increasingly longer pulses trigger an overt number of action potentials (Figure 4B, fraction of responsive neurons 1 ms 1.69 ± 0.33%, 2 ms 6.64 ± 0.92%, 5 ms 28.09 ± 4.88%, 10 ms 48.41 ± 6.04%, 20 ms 72.98 ± 7.01%, 50 ms 95.49 ± 9.02%, 100 ms 102 ± 10.35%; *p* < 0.0001 One-Way ANOVA). Within the group of responding neurons, mostly one action potential was evoked per light stimulus if the stimulus duration was 20 ms or less (Figure 4C). The average number of spikes per neuron per stimulus amounted for 1 ms to 1.05 ± 0.05, for 2 ms to 1.06 ± 0.04, for 5 ms to 1.05 ± 0.01, for 10 ms to 1.19 ± 0.02, for 20 ms to 1.57 ± 0.03, for 50 ms to 2.60 ± 0.05 and for 100 ms to 3.97 ± 0.13 (*p* < 0.0001 One-Way ANOVA). Since these results implied that a 10 ms stimulus elicited spikes with a high probability, we next investigated the response efficiency to repeated 10 ms stimulation at increasing stimulus frequencies. These experiments revealed that ChR2-positive neurons could, in principle, follow repeated stimulation up to a frequency of 50 Hz (Figure 4D). The fraction of responsive neurons was 42.41 ± 5.15% at 5 Hz, 43.19 ± 5.65% at 10 Hz, 45.37 ± 6.04% at 20 Hz and 58.70 ± 8.89% at 50 Hz (*p* = 0.29 One-Way ANOVA). The average number of spikes per neuron remained constant at stimulation frequencies of 5 Hz (1.13 ± 0.02), 10 Hz (1.01 ± 0.02), and 20 Hz (1.09 ± 0.01) (Figure 4E; *p* < 0.0001 One-Way ANOVA). Only at 50 Hz the average number of spikes per neuron was significantly increased to 1.50 ± 0.07 (5 Hz vs. 50 Hz, 10 Hz vs. 50 Hz, and 20 Hz vs. 50 Hz *p* < 0.0001 posthoc test).

In summary, we conclude from these results that using 10 ms long light pulses for optogenetic stimulation will theoretically enable us to reliably stimulate about 50% of neurons in our cortical culture model with both the physiological bursting (maximal stimulus frequency within a burst 34 Hz) and the single pulse paradigm (tonic stimulation with 0.286 Hz).

### 2.4. Spontaneous Activity Pattern Can Efficiently Be Replaced by a Physiological Burst Pattern as Well as a Non-Physiological Pulse Pattern

**Figure 5:**
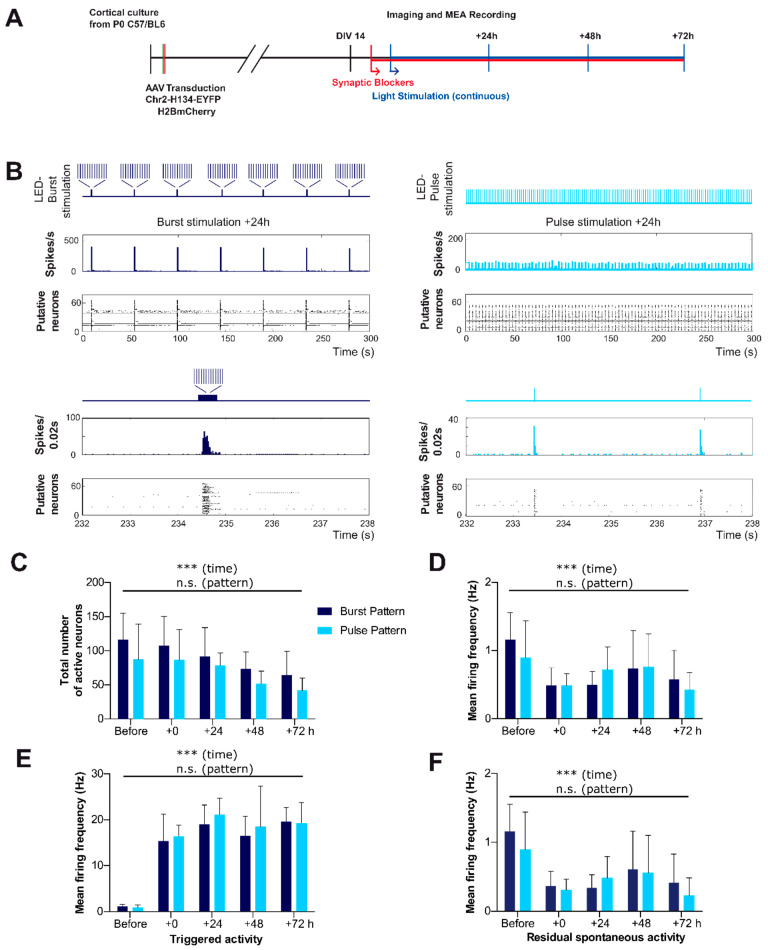
Chronic optogenetic stimulation allows alteration of neuronal activity. (**A**) Schematic overview of the experimental design. Cortical cultures from P0 C57/BL6 mice were transduced with ChR2-H134-EYFP and H2B-mCherry at DIV2 and allowed to mature for two weeks. Before and after blockade of synaptic transmission by application of glutamatergic and GABAergic receptor antagonists (CNQX, D-APV, and GBZ) at DIV14, cultures were imaged, and MEA activity was recorded for 5 min. Imaging and recording were repeated every 24 h for up to 72 h until DIV17. (**B**) Example raster plots and time histograms of cortical cultures under physiological burst stimulation (left) and non-physiological pulse stimulation (right) 24 h after the start of the chronic experiment. Note that burst stimulation elicits bursts alternated with periods of relative silence and that pulse stimulation activates neurons almost continuously at a slow pace. Lower panels: periods with single burst stimulation or two pulse stimuli at higher temporal resolution. (**C**,**D**) Comparison of the mean number of active neurons (**C**) and the mean firing frequency (**D**) before and during the chronic light stimulation with either burst and pulse stimulus pattern does not reveal a significant difference between the two stimulus paradigms (*n* = 6 cultures; *p* > 0.05 respectively). (**E**,**F**) Also, firing frequency of neurons (**E**) during and directly following the light stimulation (triggered activity within response window of 10 + 29.4 ms) as well as outside of light stimulus time points (residual spontaneous activity, (**F**) was comparable under burst and pulsed light stimulation (*p* > 0.05). Note, that the firing frequency of triggered activity significantly increased with start of light stimulation whereas the residual spontaneous activity dropped (*p* < 0.001). For calculation of activity before the optogenetic stimulation, response intervals were randomly placed and analyzed in prior non-stimulated recordings. Data points indicate mean ± SEM and *** indicates *p*-values <0.001.

For the chronic modulation of firing patterns of cortical neurons, glutamatergic and GABAergic synaptic blockers were applied to the cultures at DIV14 to enable a reliable modulation of the neuronal activity pattern during optogenetic stimulation. Together with the application of the synaptic blockers, neurons were then chronically exposed to a LED-based light stimulation that either recapitulated the physiological burst pattern or a non-physiological single pulse pattern (Figure 5A).

Importantly, both patterns consisted of the same number of stimuli (17.16 stimuli/min). MEA recordings and imaging sessions were performed as described in Figure 5A every 24 h until the end of experiments (DIV17). These experiments were performed in the continuous presence of synaptic blockers (10 µM GBZ, 10 µM CNQX, and 50 µM d-APV). The application of these synaptic blockers significantly (*p* = 0.0007 Two-Way ANOVA) reduced the frequency of neuronal firing from 1.16 ± 0.16 Hz before to 0.49 ± 0.01 in the cultures objected for subsequent burst-stimulation and from 0.89 ± 0.22 Hz to 0.49 ± 0.07 Hz in the cultures objected to pulse-stimulation experiments. As expected from the previous results regarding the establishment of the stimulus parameters (Figure 3 and Figure 4), neurons responded well to both stimulus paradigms, burst as well as pulse pattern (Figure 5B). To confirm that the general properties of neuronal activity are comparable between the two distinct stimulus paradigms, we first compared the quantitative parameters of neuronal activity between burst- and pulse-treated cultures. Cultures exposed to pulse patterns showed an equal number of active neurons when compared to cultures exposed to a chronic physiological burst pattern (Figure 5C). The number of active neurons in the burst-treated cultures was 116.5 ± 15.7 under non-stimulated control conditions, 107.3 ± 17.57 at 0 h, 91.83 ± 17.17 at 24 h, 73.67 ± 9.95 at 48 h, and 64.17 ± 14.42 at 72 h (*n* = 6 cultures, Figure 5C). In the pulse-treated cultures, the number of active neurons amounted under control conditions to 87 ± 21.24, at 0 h to 86.5 ± 18.13, at 24 h to 78.33 ± 7.57, at 48 h to 51.67 ± 7.51, and at 72 h to 41.67 ± 7.39 (*n*= 6 cultures; interaction *p* = 0.93 and pattern *p* = 0.24 Two-Way ANOVA). However, under both conditions, the number of active neurons in cultures decreased significantly over the time course of the experiments (time *p* < 0.0001 Two-Way ANOVA). Analysis of the mean frequency of all recorded extracellular spikes revealed that neuronal firing frequency also remained stable throughout the 72 h interval of the experiment and was not affected by the type of optogenetic stimulation (Interaction *p* = 0.41; Pattern *p* = 0.81 Two-Way ANOVA, Figure 5D). In cultures subjected to the burst protocol the mean firing frequency was 0.49 ± 0.01 Hz at 0h, 0.50 ± 0.08 Hz at 24 h, 0.74 ± 0.22 Hz at 48 h and 0.58 ± 0.17 Hz at 72 h (*n* = 6 cultures). In cultures subjected to the pulse protocol, the mean firing frequency was 0.49 ± 0.07 Hz at 0 h, 0.72 ± 0.14 Hz at 24 h, 0.76 ± 0.20 Hz at 48 h and 0.43 ± 0.10 Hz at 72 h (*n* = 6 cultures). Thus, quantitative analyses of activity confirmed that neuronal cultures subjected to optogenetic pulse stimulation showed the same general levels of activity as cultures, in which spontaneous activity pattern was replaced by a physiological burst pattern.

As can be seen in Figure 5B, the optogenetic stimulation protocol does not completely replace the spontaneous activity with optogenetically-induced spike, but a considerable amount of spontaneous spikes remained. Therefore, we next quantified to which extend the recorded spikes reflect optogenetic stimulation. For this purpose, we first compared the amount of triggered activity and residual spontaneous activity for the two stimulus conditions over the time course of the experiment. Spikes that occurred within a defined response window (10 ms stimulus plus the minimal interval before the next stimulus, i.e., 29.4 ms) were considered as triggered spikes. Consequently, if neurons would respond to each and every stimulation with one spike, the estimated firing frequency within the stimulus response window would theoretically be 25.4 Hz (i.e., 1/0.0394 s). In the control interval recorded in the absence of optogenetic stimulation, only a minor number of spikes (burst-triggered activity 1.16 ± 0.16 Hz, pulse-triggered activity 0.89 ± 0.22 Hz) fell randomly into this stimulus response window and were considered as catch-controls. With the start of the optogenetic stimulation, spike probability within the stimulus response window strongly increased (*p* < 0.0001 Two-Way ANOVA; *n* = 6/6 cultures) for both burst-triggered (15.37 ± 2.41 Hz at 0 h, 19.04 ± 1.70 Hz at 24 h, 16.46 ± 1.77 Hz at 48 h and 19.63 ± 1.26 Hz at 72 h) and pulse-triggered stimulation (16.34 ± 1.01 Hz at 0 h, 21.06 ± 1.50 Hz at 24 h, 18.44 ± 3.63 Hz at 48 h and 19.20 ± 1.86 Hz at 72 h) (Figure 5E). Importantly, when comparing the mean firing frequency of triggered activity between pulse- and burst- patterned stimulation, there was no significant difference (interaction *p* = 0.94, pattern *p* = 0.45 Two-Way ANOVA).

Finally, we quantified the residual spontaneous activity that still occurred outside of stimulus response windows of the patterned stimulation. As expected, the residual spontaneous activity outside of stimulus response windows was drastically reduced with the start of the patterned light stimulation time as compared to the mean frequency of all spikes (*p* < 0.0001 Two-Way ANOVA, *n* = 6/6 cultures, Figure 5F). The mean firing frequency of the residual activity for burst-patterned stimulation was 1.16 ± 0.16 Hz before, 0.37 ± 0.09 at 0 h, 0.34 ± 0.08 at 24 h, 0.61 ± 0.23 Hz at 48 h, and 0.42 ± 0.17 Hz at 72 h. The mean firing frequency of the residual activity for the pulse-patterned stimulation was 0.89 ± 0.22 Hz before, 0.31 ± 0.06 Hz at 0 h, 0.48 ± 0.13 Hz at 24 h, 0.56 ± 0.22 at 48 h, and 0.23 ± 0.1 at 72 h. However, again, neurons exposed to burst stimulus patterns showed the same level of residual spontaneous activity as neurons exposed to pulse patterns (interaction *p* = 0.63, pattern *p* = 0.56). From the observed optogenetically-induced spikes, we calculated that, on average, 11.75 ± 1.51 spikes were induced per burst at 0 h, 13.12 ± 1.17 spikes at 24 h, 15.15 ± 4.07 spikes at 48 h, and 11.39 ± 6.7 spikes at 72 h (data not shown). These results emphasized that our stimulation protocol could reliably emulate the properties of the physiological burst activity (13 spikes per burst at DIV 14).

In summary, these analyses demonstrated that neurons in ChR2-expressing cultures responded quite reliable and to a comparable extent to the burst- and the pulse-like LED-based optogenetic stimulation. Thus, continuous optogenetic stimulation allowed the effective control and manipulation of the neuronal firing of genetically modified neurons under blockade of glutamatergic and GABAergic synaptic communication and thereby to reinstate either bursting activity resembling the physiological, spontaneous activity, or a pulse-like pattern with a similar number and frequency of spikes but non- physiological pulse-like pattern.

### 2.5. Manipulation of Firing Pattern Alters Neuronal Gene Expression and Neuronal Survival

**Figure 6:**
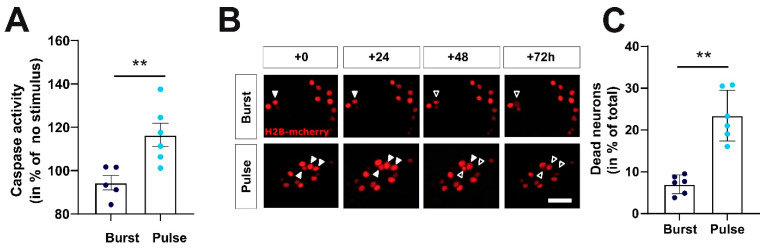
Optogenetically provoked changes in firing patterns critically regulate caspase activity and neuronal cell death rates during late development. (**A**) After 48 h of continuous optogenetic stimulation with the burst pattern caspase activity was lower as compared to optogenetic stimulation with pulse pattern (*n* = 5/6 cultures; *p* < 0.01). (**B**) Representative images of H2B-mCherry positive nuclei subjected to optogenetic burst and pulse stimulation over the time course of the experiment. Filled arrows mark cells that died during the time course of the experiment. Unfilled arrows mark apoptotic or dead neurons, respectively. (**C**) Statistical analysis revealed that significantly more neurons are dead after 72 h if cultures were stimulated with pulse pattern compared to stimulation with physiological burst pattern (*n* = 6/6 cultures, *p* = 0.002). Scale bar represents 50 µm. ** indicates *p*-values <0.01.

After we verified that the stimulation protocols were suitable to substitute spontaneous activity with either physiological, burst-like activity or non-physiological, pulse-like activity, we finally determined how these different stimulation paradigms affect neuronal survival. To compare their effect on neuronal apoptosis, we performed a luminescent assay that measures caspase-3 and -7 activities after 48 h of continuous optogenetic stimulation (Figure 6A).

Upon addition of the luminogenic caspase-3/7 substrate at DIV16, cultures exposed to continuous burst stimulation for 48 h showed significantly (*p* < 0.01 students t-test) lower luminescence values as cultures exposed to pulse stimulus paradigm indicating lower amounts of caspase activity present upon burst stimulation (Figure 6A). The caspase activity, normalized to non-light controls with synaptic block, amounted to 94.39 ± 3.3% (*n* = 5 cultures) upon burst stimulation and was significantly higher upon pulse stimulation (116.4 ± 5.4%; *n* = 6 cultures). To control for potential unspecific effects of the light stimulation per se, cultures devoid of ChR2-expression were stimulated with either the burst- or the pulse-like stimulation for 48 h, and caspase activity was subsequently assessed. Chronic light stimulation in absence of ChR2 expression did not significantly (*p* = 0.34 One-Way ANOVA) alter caspase activity, neither when the burst-like stimulus paradigm was applied (90.57 ± 3.65% *n* = 6 cultures, posthoc test control vs. burst *p* = 0.37), nor with the pulse-like stimulation (100.7 ± 7.21% *n* = 6 cultures, posthoc test control vs. pulse 0.99).

In addition, we performed a longitudinal analysis of cell survival based on the H2B-mCherry nuclear signals of neurons throughout the experiment (DIV14-17; Figure 6B). For this longitudinal analysis, we analyzed nuclear H2B-mCherry signals of 271.8 ± 14.6 neurons per culture (average number of H2B-positive neurons in the field of view covering roughly the 120-electrode MEA array at DIV14). Cell death rates were compared at the end of the experiment at DIV17, thus after 72 h of continuous patterned optogenetic light stimulation. In line with the preceding elevation of caspase activity at DIV16, we observed that the fraction of dying neurons in cultures stimulated with the burst-like protocol (7.01 ± 0.91%; *n* = 6 cultures) was significantly (Mann–Whitney test *p* = 0.002) lower than in the cultures exposed to the pulse-like optogenetic stimulus protocol (23.45 ± 2.47%; *n* = 6 cultures, Figure 6B). In addition, in line with the results from the caspase assay, the fraction of dead neurons during optogenetic light stimulation was increased compared to control experiments under synaptic blockade but with absent light stimulation (4.13 ± 2.7%, *n* = 4 cultures) when the pulse-like stimulus pattern was applied (One-Way ANOVA *p* < 0.0001, posthoc test control vs. pulse *p* < 0.0001) but remained comparable when the burst-like stimulus pattern was applied (posthoc test control vs. burst *p* = 0.62). Thus, optogenetic stimulation with a protocol that resembles physiological activity is significantly more effective in protecting neurons from neuronal apoptosis than a stimulus protocol that delivers a similar number of action potentials with a non-physiological distribution.

**Figure 7:**
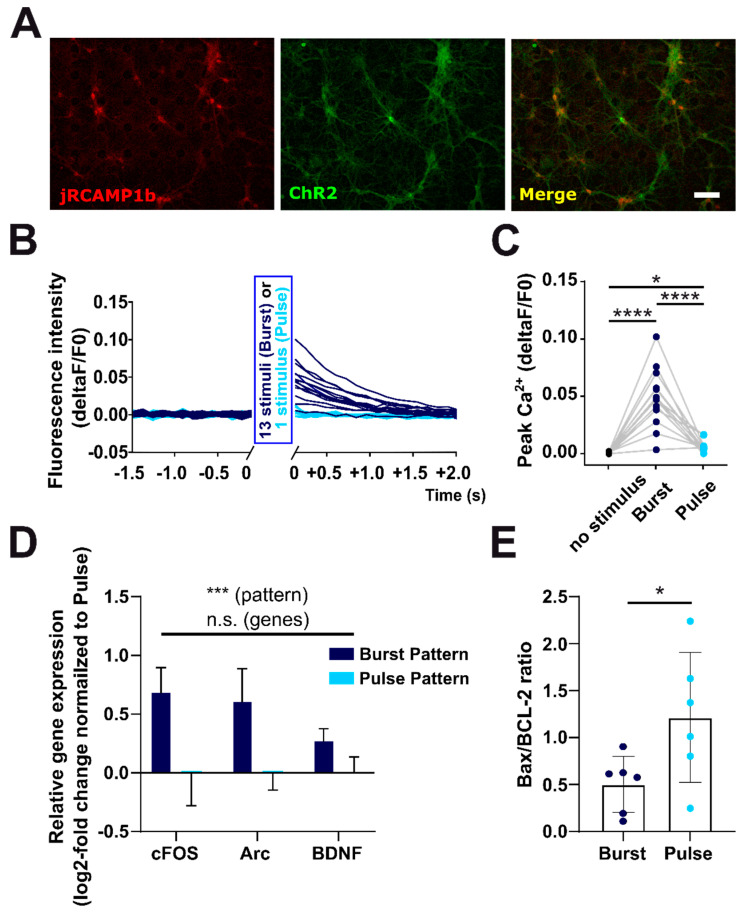
Activity-pattern-dependent regulation of neuronal Ca^2+^ and gene expression precedes differences in neuronal cell death. (**A**) Representative images show the jRCAMP1b and ChR2-signal as well as the merged signal in cortical neurons at DIV 14. (**B**) Average Ca^2+^ response of ChR2-positive neurons after a single pulse-like stimulus (light blue) and after a burst-like stimulation (dark blue, traces aligned to the last stimulus in the burst). The control intervals before the stimuli were aligned to the onset of the stimulus or the onset of the first stimulus in the burst, respectively. Ca^2+^ signals were acquired by imaging with the red Ca^2+^ indicator jRCAMP1b. (**C**) Peak intensity of optogenetically induced calcium response was significantly smaller upon pulse stimulation as compared to burst stimulation (*n* = 14 cells; *p* < 0.01). (**D**) Comparison of gene expression between cultures subjected to burst or pulse stimulus pattern for 48 h showed significant differences in regulation of classical activity regulated targets (BDNF, cFOS and arc) (*n* = 6/cultures; *p* < 0.001). (**E**) In line with a lower survival rate after 72 h, cortical neurons exposed to non-physiological pulse stimulus pattern present a significantly higher Bax/Bcl2 ratio already after 48 h of optogenetic stimulation (*n* = 6/6 cultures; *p* < 0.05). Scale bar represents 50 µm. Data points indicate mean ± SEM and *, *** and **** indicate *p*-values <0.05, 0.001, and 0.0001, respectively.

In order to get the first insight into cellular and molecular mechanisms that underlie the differential effects of the two different optogenetic stimulation paradigms on cell survival and apoptosis rates, we next investigated the effect of the different stimulus patterns on the intracellular Ca^2+^ levels in cortical neurons by simultaneous calcium imaging with the red indicator jRCaMP1b (Figure 7A). During the optogenetic light stimulus, the Ca^2+^ signal was obscured by concomitant photonic LED light stimulation. Yet, both stimulus types, pulse-like as well as burst-like stimulation, resulted in a notable increase in intracellular Ca^2+^ that outlasted the LED pulse for optogenetic stimulation (Figure 7B). Thus, Ca^2+^ responses could reliably be determined directly after the end of the optogenetic stimulation with a maximal delay of 0.125 s after the end of the stimulation. For pulse-like stimulation the average stimulus-induced peak Ca^2+^ response (maximal deviation ΔF/F0, Figure 7C) was 4.9 × 10^−3^ ± 1.4 × 10^−3^ (*n* = 84 stimulus responses from 14 cells), which was significantly higher (repeated-measure ANOVA *p* < 0.0001, posthoc test pulse vs. no-stimulus *p* < 0.05) than the no-stimulus responses (0.4 × 10^−3^ ± 0.2 × 10^−3^, *n* = 84 stimulus responses from 14 cells). The average peak Ca^2+^ response to burst stimulation (48.4 × 10^−3^ ± 6.5 × 10^−3^, *n* = 84 stimulus responses from 14 cells) was also higher than no-stimulus responses (posthoc test no stimulus vs. burst *p* < 0.0001), and the response to burst-like stimulation was also higher when compared to pulse (posthoc test Pulse vs. Burst *p* < 0.0001).

To further characterize the stimulus-induced intracellular Ca^2+^ transients, we calculated the area under the curve for each stimulus-response. Here, burst-like stimulation also caused a significantly larger response than a singular pulse-like stimulation (no stimulus 0.046 ± 0.015 a.u.; Burst 2.75 ± 0.14 a.u.; Pulse 0.176 ± 0.03 a.u.; *n* = 120 stimulus responses each from 20 cells, 2-Way ANOVA *p* < 0.0001 posthoc test no stimulus vs. burst *p* < 0.0001, burst vs. pulse *p* < 0.0001). As a burst-like stimulation consisted of 13 individual stimuli, we also compared the average calcium response of one burst-like stimulation (2.75 ± 0.14 a.u) to the equivalent that would be evoked by 13 stimuli in the pulse paradigm. This value amounts to 2.28 ± 0.931 a.u. and is not significantly smaller (posthoc test burst vs. 13xpulse *p* = 0.36), suggesting that the Ca^2+^ response upon burst stimulation represents only a summation of the individual stimulus-induced Ca^2+^ transients. In summary, this experiment revealed that burst-like stimulation generated a considerably higher and longer-lasting intracellular Ca^2+^ transient than a single pulse-like stimulation, which could mediate the distinct downstream consequences of the different optogenetic stimulation paradigms on the cellular level.

To investigate the regulatory mechanisms that underlie these activity-pattern-dependent differences in neuronal survival rates on the molecular level, we performed quantitative real-time PCR of some classical activity and apoptotic regulatory elements upon 48 h of continuous burst-like or pulse-like optogenetic light stimulation. Using this protocol, we analyzed the relative expression of the transcription factors c-Fos and Arc [38], classical markers for neuronal activity, the brain-derived neurotrophic factor (BDNF [39], as well as Bax and BCL-2, important regulators of apoptosis [40]. Our results demonstrated that the expression levels for all of these candidate genes differed significantly upon a 48 h stimulation with the physiological burst or the non-physiological pulse pattern (Two-Way ANOVA stimulus pattern *p* = 0.0008, *n* = 6/6 cultures) (Figure 7D,E). In line with a stronger pro-survival effect of the optogenetic burst stimulation and a higher peak calcium response, the classical immediate early genes c-Fos, Arc and the neurotrophic factor BDNF were significantly upregulated (Two-Way ANOVA stimulus pattern *p* = 0.0005 genes *p* = 0.70 interaction *p* = 0.35) at 48 h hours after start of stimulation with burst pattern (Relative expression c-Fos: Burst 0.69 ± 0.22 Pulse 0.00 ± 0.28; Arc: Burst 0.62 ± 0.29 Pulse 0.00 ± 0.15; BDNF: Burst 0.28 ± 0.11 Pulse 0.00 ± 0.14; *n* = 6/6 replicates from 3 cultures each) (Figure 7D).

Most interestingly, we could, at the same time, find a significantly (*p* = 0.02 students t-test) lower expression of the pro-apoptotic transcription factor Bax in cultures subjected to the physiological burst pattern (relative expression Bax: Burst −0.19 ± 0.05 Pulse 0.00 ± 0.0.04; *n* = 6/6 replicates from 3 cultures each). In contrast, the expression of the anti-apoptotic factor BCL-2 was not significantly altered (*p* = 0.28 students t-test) by the optogenetic stimulus paradigm (Relative expression BCL-2: Burst 0.21 ± 0.10 Pulse 0.00 ± 0.15; *n* = 6/6 replicates from 3 cultures each). As a critical measure for the regulation of neuronal cell death or survival, we calculated the Bax/BCL-2 ratio (Figure 7E) for each culture, and in line with a prospective higher cell death rate at 72 h this ratio was significantly increased (*p* = 0.04 students t-test) in cultures subjected to the non-physiological pulse-like pattern (1.21 ± 0.28; *n* = 6 replicates from 3 cultures) compared to burst-like pattern (0.50 ± 0.12; *n* = 6 replicates from 3 cultures). Thus, the reduced Bax/BCL-2 ratio may in part explain the reduced cell death rates upon burst stimulation.

In summary, our results demonstrate that optogenetic stimulation with a stimulus pattern that replicated physiological burst firing decreased the rate of neuronal cell death as compared to the non-physiological pulse pattern potentially by an activity-pattern dependent upregulation of the neurotrophin BDNF and/or downregulation of the pro-apoptotic transcription factor Bax.

## 3. Discussion

The main observations of the present study are as follows: (i) Physiological and non-physiological patterns of neuronal activity can be reinstalled in ChR2-expressing neurons in cell-cultures silenced by GABAergic and glutamatergic blockers. (ii) Reinstalling a physiological burst pattern reduced the number of apoptotic neurons, in contrast to a non-physiological stimulation paradigm using the same number of stimuli at equidistant repetitions. (iii) Burst pattern stimulation-induced larger Ca^2+^ transients, a higher expression of BDNF, and decreased Bax/BCl-2 ratio, which can contribute to the anti-apoptotic effect of this physiological stimulation paradigm. In summary, our results indicate that the temporal pattern of neuronal activity in the immature brain can directly influence the regulation of apoptosis in neurons during development and highlight the importance of highly correlated bursting activity for the structural development of the central nervous system.

The transition from sparse, uncorrelated firing of neurons to highly-correlated bursting activity of neurons is a general and well-conserved developmental feature of cortical networks before neuronal activity enters the mature phase that is again dominated by decorrelated network activity [41,42,43]. Interestingly, exactly this transition period of correlated electrical activity coincidences with the peak of cell death rates in postnatal development in vivo [17,23,31] as well as in vitro [13,36]. Primary cortical cultures bear the advantage of high accessibility to genetic and pharmacological manipulation and controllability of electrical and cellular features down to the cellular level [37]. For these reasons, we have chosen primary cortical cultures as a model to closely investigate the role of activity pattern-dependent regulation of developmental apoptosis in the present study.

Pharmacological population-based approaches in previous studies have further shown that blockade or modulation of network activity levels leads to changes in cell death rates in developing cortical networks in vivo and in vitro [6,10,11]. Yet, it remained unclear if network-independent changes in cell-intrinsic activity levels could be neuroprotective and what the significance of synaptic (i.e., NMDA- and AMPA- or GABA-A receptor-mediated transmission) and extrasynaptic inputs for the survival of developing neocortical neurons is [44,45]. In line with previous studies [6,10,11,16], longitudinal imaging of nuclear H2B-mCherry signals in the present study confirmed that chronic blockade of neuronal firing in naive primary cortical networks over the course of multiple days leads to a significant increase in cell death rates throughout development. However, inhibition of glutamatergic and GABAergic inputs is critical for the survival of neurons only in the early but not the late developmental period. Pharmacological blockade of synaptic inputs, in general, is inevitably associated with changes in the spiking behavior of neurons. In general, the blockade of glutamatergic AMPA receptors reduces neuronal firing rates within a short period of time due to the block of the main synaptic input signals. However, subsequently, firing rates can be homeostatically regulated if a glutamatergic block affects networks for a longer period [46]. Contrariwise, blockade of neuronal firing leads to changes in synaptic inputs via synaptic scaling [47]. Accordingly, in our studies, a considerable amount of spiking activity was maintained in the presence of the receptor blockers CNQX, APV, and GBZ. This mutual dependency of synaptic activity and action potential firing is also emphasized by the absence of additive effect of combined application of TTX and synaptic blockers on cell death rates in the present study and hampers the interpretation of results from previous studies with pharmacological or electrical manipulations of cortical network activity. Intriguingly, after the optogenetic application of sufficient levels of neuronal activity, the amount of residual, unprovoked spikes are considerably reduced, suggesting a general contribution of homeostatic metaplasticity in the sustained spontaneous activity after the chronic synaptic blockade. This suppression of unprovoked activity under pharmacological suppression of glutamatergic and GABAergic synapses allows us to reliably reinstate physiological and non-physiological spike patterns in transgenic cortical cultures using LED-based optogenetic stimulation. Transgenic expression of light-gated ChR2 allows the direct optogenetic activation of targeted neurons [48]. At the same time, it allows the combination with other genetic or pharmacological tools, e.g., expression of a fluorescent histone-tag to visualize the viability state of the neurons and GABAergic and glutamatergic blockers to isolate the targeted neurons synaptically. The installation of LED-based light stimulation in the incubator to perform long-term chronic stimulations ensured optimal culturing conditions [49] and enabled us to effectively and continuously modulate neuronal activity patterns over the course of multiple days. The efficiency of the LED-based stimulation was tested by single-cell patch clamp experiments, in which the experiments focused on the response properties of visually-identified ChR2 positive neurons and by extracellular MEA recordings, which unbiasedly revealed the overall response probability in the recorded neuronal population. Both methods verified that short LED-based light stimulation with a stimulus duration of 5–10 ms efficiently evoked spikes in synaptically isolated neurons and that repeated light stimulation is possible up to a stimulus frequency in the gamma range. Repeated MEA recordings during the course of the longitudinal experiments were performed to ensure that continuous, patterned LED-based stimulation caused a lasting alteration of the neuronal activity patterns and that the different stimulation patterns evoked a comparable amount of total spikes in a comparable number of neurons.

As our electrophysiological recordings revealed that the ChR2-positive neurons can reliably follow the used stimulation patterns and that this optogenetic stimuli dominated the neuronal activity throughout the dissociated neuronal culture, we were able to reinstall physiological levels of neuronal activity with optogenetic stimulation. Based on our central hypothesis that the pattern of neuronal has differential effects on apoptosis, we generated two distinct stimulation patterns that share the same number of stimuli but had either a non-physiological evenly distributed appearance or a burst pattern that resembles the temporal structure of the recorded activity. Notably, we observed that more neurons survive under the physiological burst-like stimulation as compared to the cultures exposed to the non-physiological pattern. This suggests, on the one hand, that cell-intrinsic neuronal excitation can directly influence cell survival and death rates without the need for synaptic inputs from the surrounding network and, on the other hand, that not only the number of spikes but the patterning of the activity directly determines the survival rate in developing neurons. Here, it is important to note that with the blockade of ionotropic glutamatergic and GABAergic receptors by CNQX, APV, and GBZ the presynaptic release of neurotransmitters and the postsynaptic response of glutamatergic and GABAergic G protein-coupled receptors remain unaffected. Thus, downstream signaling of these receptors (e.g., mGluRs) could contribute to the neuroprotective effect of optogenetically induced burst-firing [50]. On a cellular level, patterned, correlated high-frequency activity is strongly associated with differentiation and neuronal growth [51], the release of neurotrophic factors [52], and changes in synaptic plasticity [53,54]. And accordingly, our further experiments indeed demonstrated that the physiological burst-like activity led to larger Ca^2+^ transients, which may serve as a main second messenger for further neuroprotective processes [55,56,57]. The Ca^2+^ transients upon burst-like optogenetic stimulus are reliably back-regulated within 2 s and thus resemble typical Ca^2+^ transients observed in vitro and in vivo under physiological activity levels in the immature central nervous system [58,59].

In addition to the increased Ca^2+^ concentrations, PCR-based analysis of gene expression revealed that only burst-like stimulation was sufficient to increase expression levels of cFos and Arc, typical factors that indicate the activity-dependent upregulation of gene expression [60]. Of particular relevance was our observation that optogenetic burst stimulation also leads to an increased BDNF gene expression. This result is in line with the previous findings that optogenetic stimulation of neurons results in a significant and neuroprotective release of neurotrophic factors despite the absence of synaptic communication [61] and that BDNF secretion requires sufficient and sustained Ca^2+^ elevations [62,63]. As BDNF is one major anti-apoptotic cytokine [64], the enhanced BDNF secretion can contribute to the anti-apoptotic effect of burst stimulation. Our PCR-based analysis of gene expression also revealed a downregulation of the pro-apoptotic factor Bax, while the anti-apoptotic factor BCL-2 was unaffected. The decreased Bax/BCL-2 ratio is a hallmark of anti-apoptotic states [65,66] and is probably causally contributing to the decreased apoptosis rates under burst-like stimulation. Accordingly, our experiments also provide evidence that only burst-like stimulation directly reduces the caspase activity, as indicated by the lower cleavage of the luminogenic caspase-3/7 substrate.

The multilayered and complex regulation of neuronal apoptosis on the cellular and molecular levels is intensively investigated [3,4,5,8,40,67], and the contribution of neuronal activity to this process has already been acknowledged [9,10,12,13,14,15,23]. However, to our knowledge the present study adds for the first-time evidence for a potential direct activity-dependent regulation of classical apoptosis regulators of the BCL-family, i.e., the downregulation of the pro-apoptotic Bax and the lower Bax/BCL-2 ratio in burst-stimulated cultures. This mechanism could enable network-independent cell fate decisions on the single neuron level based on the presence or absence of critical activity patterns during development [7,17,21,23]. And even more important, we demonstrated for the first time that the pattern of neuronal activity directly influences neuronal apoptosis, with a pattern resembling the coincident burst firing typical for immature networks promoting a substantial anti-apoptotic effect. Interestingly, high-frequency bursting activity is a highly conserved hallmark feature of developing cortical networks across mammalian species from rodents to primates [42,43], and their physiological and clinical importance is highlighted by the fact that the absence of these high-frequency activities correlates in human EEG recordings is commonly associated with severe neurological and forthcoming maldevelopment of affected individuals [68,69].

## 4. Material and Methods

### 4.1. Primary Neuronal Cultures

Experiments were performed in primary cortical neurons cultured from newborn (postnatal day 0) mice (C57BL6J/N). All animal experiments in this study were conducted in accordance with National and European (86/609/EEC) laws for the use of animals in research and were approved by the local ethical committee (Landesuntersuchungsamt Rheinland-Pfalz 23.177–07/G 10-1-010 and G20-1-006). After decapitation, brains were transferred to ice-cold Ca^2+^- and Mg^2+^-free HBSS (Gibco, Invitrogen, Carlsbad, CA, USA) supplemented with penicillin and streptomycin (50 units/mL), sodium pyruvate (11 mg/mL), glucose (0.1%), and HEPES (10 mM). Cortical cells were dissociated via trypsin incubation for 20 min at 37 °C and DNAse digestion at RT. After blocking trypsinization by washing steps with HBSS, Minimal Essential Medium (MEM, Gibco, Thermo Fisher Scientific, Waltham, MA, USA) supplemented with 10% horse serum, and 0.6% glucose was added. Next, the cells were mechanically dissociated via repetitive pipetting through fire-polished glass pipettes with declining diameters. Following cell counting, cells were seeded on polyethyleneimine- or polyornithin coated multi-electrode arrays (MEAs, 120MEA100/30iR-Ti-gr, Multi-Channel Systems), glass coverslips, or cell culture imaging chambers (IBIDI, Graefelfing, Germany) (density: 2500 cells/mm^2^). After 30 min the medium was exchanged for a medium consisting of Neurobasal medium (Gibco) supplemented with 2% B27 (Gibco) and 1 mM L-glutamine. Cells were cultivated at 37 °C in humidified carbogen (95% air; 5% CO_2_). Primary neurons were transduced after 1 day in culture with recombinant AAV (rAAV1/2; appr.1-3x104 copies per cell) carrying either or a combination of pAAV-hSyn-H2B: mCherry, pAAV-CaMKIIa-hChR2(H134R)-EYFP or pAAV.Syn.NES-jRCaMP1b.WPRE.SV. pAAV.Syn.NES-jRCaMP1b.WPRE.SV40 was a gift from Douglas Kim and GENIE Project (Addgene plasmid # 100851; http://n2t.net/addgene:100851 (accessed on 1 June 2021); RRID:Addgene_100851) and pAAV-CaMKIIa-hChR2(H134R)-EYFP was a gift from Karl Deisseroth (Addgene plasmid # 26,969; http://n2t.net/addgene:26969 (accessed on 1 June 2021); RRID:Addgene_26969). Upon AAV based transduction of primary neurons with H2B-mCherry and ChR2 at DIV2 expression levels of transgenes had reached plateau levels around DIV14. After 2 days, 5 µM AraC was added to the medium to inhibit glial cell proliferation. After 1 week, half of the medium was exchanged.

### 4.2. MEA Recordings

Extracellular electrical recordings were performed as described previously [37]. In short, cells were cultured on MEAs containing 120 planar extracellular titanium nitrite electrodes with 4 internal references (120MEA100/30iR-Ti-gr, Multi-Channel Systems, Reutlingen, Germany). MEAs had an electrode diameter of 30 μm and an interelectrode spacing of 100 or 200 μm. Signals from 120 recording electrodes were recorded with MC_Rack software in a MEA 2100 system (Multi-Channel Systems) at a sampling rate of 50 kHz and high-pass filtered at 200 Hz. Spikes were detected using a negative threshold-based detector set to a threshold of 7× the SD of the noise level (MC_Rack, Multi-Channel Systems). Electrophysiological recordings and imaging were performed in a culture medium. The temperature was maintained at 37 °C by a temperature controller (TC02, Multi-Channel Systems). Spike datasets from all electrodes recorded for 10 min were imported into Plexon software for analysis of single units using a spike sorting algorithm. Plexon’s spike sorting methods work in feature space, characterizing the essence of the waveform by using certain calculated features instead of the entire raw waveform (Projections onto Principal Components (PCA), waveform heights at chosen times). The applied automatic sorting method ‘Valley seeking’ finds neurons and assigns waveforms to neurons by comparing those features that were most variable in all waveforms. The algorithm was applied to inter-point distances to automatically determine the number of clusters and the cluster memberships (waveforms). If single units fired ≥2 times within the recording period, units were counted as active neurons. Average firing frequencies were calculated as the arithmetic mean of individual firing frequencies of all identified units. Burst detection, as well as analysis of stimulus efficiency, was calculated with custom-made analysis routines in Matlab. Only spike trains consisting of 5 or more spikes with an interspike interval of <100 ms were considered burst [70].

### 4.3. Optogenetic Light Stimulation

For continuous patterned light stimulation of dissociated cortical culture LED arrays controlled by a small microcontroller were custom built. Herewith, multiple cultures (e.g., MEAs or 8-well plates or coverslips) could also be stimulated simultaneously with different stimulation protocols. For the microcontroller, an Arduino Uno (Arduino, Somerville, MA, USA), a microcontroller board based on the ATmega328P, was used. The used LEDs were high-power LEDs emitting at 470 nm to activate neurons transduced with ChR2-H134R (Lumileds LXML-PB01-0040, LUXEON Rebel 470 nm Bleu High-Power LED) with an average light intensity of approximately 8–9 mW/mm^2^ (PM100D, Thorlabs, Newton, MA, USA). For the continuous patterned light stimulation, a 12-well LED array was used, in which LEDs were arranged in a 3 by 4 grid. Each set of 3 LEDs was wired in series. Each series was connected to its own LED driver. The LED driver used was the small-size single-output constant current FemtoBuck Led Driver, developed by Sparkfun. Each LED driver, in turn, was connected to an individual digital pin on the Arduino, controlling each series of LED’s on/off state (blinking frequency), pulse width, and light intensity in a programmed protocol. The LED array fitted in a small incubator, maintaining physiological conditions and thus allowing for long-term stimulation. The LED array closed off entirely with a lid on top and in front and the 3-series LEDs were separated by light-impregnatable walls making each partition light-tight from incoming light outside the box or from other partitions.

For stimulation of cultures during simultaneous MEA-based electrical recordings or optical imaging, a single LED circuit was built, using also a LUXEON Rebel 470 nm Bleu High-Power LED and an Arduino Uno, that fitted under the head stage of the MEA recording system. The Arduino Uno was connected to the interface board of the MEA-setup so that the LED on and off times could be registered. Because the light intensity of the LED was relatively low, especially compared to laser light, no light artifacts due to a photoelectric effect occurred.

### 4.4. Whole Cell Patch Clamp Experiments

For patch clamp experiments, the coverslips with the cultured cells were transferred at DIV14 into ACSF consisting of (in mM) 126 NaCl, 26 NaHCO_3_, 1.25 NaH_2_PO_4_, 1 MgCl_2_, 2 CaCl_2_, 2.5 KCl, 10 glucose, equilibrated with 95% O_2_/5% CO_2_ (pH = 7.4) and at an osmolarity of 316 mOsm. In the experimental setup, cells were constantly perfused with oxygenated ACSF at a rate of 2 mL/min. All experiments were performed at 31 ± 1 °C. Neurons were visualized on the coverslips using an inverted microscope (BX51WI, Olympus, Hamburg, Germany) equipped with a CCD camera (VX45, Optronics, Goleta, CA, USA) connected to a video monitor (PVM-145E, Sony, Weybridge, UK). Epifluorescence was used to identify ChR2-H134-EYFP and H2BmCherry-positive cells before the recording. The patch electrode, as well as the stimulation LED, were positioned using electric manipulators (SM-1, Luigs and Neumann, Ratingen, Germany). The stimulation LED (LUXEON Rebel 470 nm Bleu High-Power LED, details see Optogenetic Light Stimulation) was positioned above the recording chamber at a distance of ca. 10 mm and an angle of 30°. Patch clamp recordings were conducted with a discontinuous voltage clamp/current clamp amplifier (SEC05L, NPI, Tamm, Germany), connected to a standard personal computer via a digital analog converter (LIH1600, HEKA Electronics, Lambrecht, Germany). Stimulation protocols, data recording, and posthoc data analysis were performed using TIDA software (TIDA 5.25, HEKA, Reutlingen, Germany). Patch electrodes and application pipettes were pulled from borosilicate glass capillaries (GB200F-8, Science Products, Hofheim am Taunus, Germany) using a vertical electrode puller (Model PP-830, Narishige Co., Tokyo, Japan). The patch electrodes (resistance 3 to 5 MΩ) were filled with a K-gluconate based pipette solution containing 10 mM Cl- (128 K-gluconate, 4 KCl, 1 CaCl2, 4 NaCl, 11 EGTA, 10 K-HEPES, pH adjusted to 7.4 with KOH and osmolarity to 300 mOsm with sucrose). Whole-cell recordings were performed in current clamp mode, while stimulating the culture with blue LED light at different pulse frequencies and pulse durations to be able to analyze the induction of action potentials. Throughout these experiments, 10 µM CNQX and 60 µM APV were added to the bath solution to inhibit postsynaptic potentials and to isolate the direct postsynaptic responses of optogenetic stimulation. Some experiments were performed in presence of 1 µM TTX to assess the significance of optogenetically induced depolarizations above possible electrode current artifacts.

### 4.5. Optical Imaging

Imaging of neurons on MEAs was performed with an upright microscope (OlympusBX61WI) equipped with a Hamamatsu Orca R2 C10600 CCD camera and a MT-20 light source to ensure that no cells were masked by an electrode. Images were subsequently analyzed with custom-designed ImageJ macros.

Long-term neuronal imaging was performed within a custom-built incubator that allows simultaneous electrophysiological recording and microscopic observation of cortical neurons over the course of several days in incubator-like conditions. In addition, a subset of experiments was performed in the presence of the caspase substrate NucView488 (1.5 µM, Biotium, Freemont, CA, USA) to allow the real-time detection of apoptosis.

Quantification of cell death was performed based on nuclear H2B-mCherry signal, which allows for ongoing assessment of cell death of individual cells throughout the entire duration of the experiments. In more detail, cell death rates of neuronal cultures were quantified by a 3-step process. First cultures were imaged at DIV14 and subsequently 24, 48 and 72 h later. Then, the total number of cells was counted by using an ImageJ macro, which finds coordinates of H2B-positive cells at DIV14 and at DIV17 (+72 h), respectively. To rule out artifacts, fluorescent images of the culture at 24 and 48 h were carefully studied. Finally, the coordinates of these 2 time points were compared. If nuclear H2BmCherry-signal disappeared, the respective neuron was considered as dead. In addition, if a cell had not disappeared completely, but clear hallmarks of cell death were visible, such as chromatin condensation and/or DNA fragmentation, it was also considered a dead cell.

### 4.6. Combined Calcium Imaging and Optogenetic Stimulation

Combined imaging and optogenetic stimulation were performed similarly as described in the preceding sections. Consecutive imaging before, while, and after samples were illuminated by an LED mounted from below was done with an imaging frequency of 8 Hz and respective filter sets. Repeated stimulation with the different stimulus patterns was applied. Neurons co-expressing NES-jRCaMP1b and CaMKIIa-hChR2(H134R)-EYFP were chosen for comparative analysis of calcium response to the different stimulus paradigms. Image sequences were subsequently analyzed with ImageJ and Matlab.

### 4.7. Caspase Glow Assay

Caspase activity was measured by a luminescent Caspase-Glo^®®^ 3/7 Assay (Promega, Madison, WI, USA) after 48 h of optogenetic stimulation (DIV16). This homogeneous, luminescent assay provided a luminogenic caspase-3/7 substrate, which contained the tetrapeptide sequence DEVD, in a reagent optimized for caspase activity, luciferase activity, and cell lysis. The protocol was performed according to the manufacturer’s guidelines. In short, equal amounts of Caspase-Glo^®^ 3/7 reagent and PBS were added to the cells upon removal of the medium. Buffer and cells were mixed and incubated on the samples for 30–45 min. Luminescence was measured with a Tecan infinite M1000 plate reader (Tecan, Maennedorf, Switzerland) and normalized to non-stimulated light controls for statistical analysis.

### 4.8. Real-Time PCR

Total RNA from primary cortical cultures was isolated after 48 h of optogenetic stimulation (DIV16) using the RNeasy Mini Kit (Qiagen, Venlo, Netherlands). mRNA was reverse transcribed using the Transcriptor High Fidelity cDNA SynthesisKit (Roche Applied Science, Basel, Switzerland). qRT-PCR was performed using the iTaq Universal SYBR Green Supermix (Bio-Rad, Hercules, CA, USA) well as primers designed with the Universal Probe Library in a LightCycler 1.5 System (all Roche Applied Science). Primer sequences (in 5′-3′orientation) of the target gene were as follows: c-Fos CTC CTG TCA ACA CAC AGG ACT and GCTGTCACCGTG GGGATAAA, Arc GTGAGCTGAAGCCACAAATCC and TCTTCACTGGTATGAATCACTGG, BDNF (Exon4) CCACCAACAGTCATCATGGA and GCTGCCTTGATGTTTACTTTGA, Bax CCACCAACAGTCATCATGGA and CGTCCTCGAAAAGGGCTAA, BCL-2 GTACCTGAACCGGCATCTG and GCTGAGCAGGGTCTTCAGAG and β-actin, TGACAGGATGCAGAAGGAGA and CGCTCAGGAGGAGCAATG. The qRT-PCR crossing points were used for relative quantification based on the ΔΔCt-method using the StepOne software (version 2.3), and β-actin was used as a reference gene [71].

### 4.9. Statistics

Values were given as mean values ± SEM. All statistical tests were performed using GraphPadPrism (GraphPad, LaJolla, CA, USA) or appropriate Matlab functions. Comparisons between multiple groups were performed with a One-Way ANOVA or a repeated-measure ANOVA followed by a Tukey’s Multiple Comparison Test for posthoc analysis, or a two-way ANOVA followed by a Sidaks Multiple Comparison Test, when applicable. Comparisons between two groups were performed with students un-paired t-test or non-parametric Mann–Whitney tests, when applicable. Significance was considered at *p* values < 0.05.

## Data Availability

Data is contained within the article.

## References

[1] Kuan C.Y., Roth K.A., Flavell R.A., Rakic P. (2000). Mechanisms of programmed cell death in the developing brain. Trends Neurosci..

[2] Dekkers M.P., Barde Y.A. (2013). Programmed cell death in neuronal development. Science.

[3] Wong F.K., Marín O. (2019). Developmental cell death in the cerebral cortex. Annu. Rev. Cell Dev. Biol..

[4] Yamaguchi Y., Miura M. (2015). Programmed cell death in neurodevelopment. Dev. Cell.

[5] Pfisterer U., Khodosevich K. (2017). Neuronal survival in the brain: Neuron type-specific mechanisms. Cell Death Dis..

[6] Blanquie O., Kilb W., Sinning A., Luhmann H.J. (2017). Homeostatic interplay between electrical activity and neuronal apoptosis in the developing neocortex. Neuroscience.

[7] Kirischuk S., Sinning A., Blanquie O., Yang J.W., Luhmann H.J., Kilb W. (2017). Modulation of neocortical development by early neuronal activity: Physiology and pathophysiology. Front. Cell. Neurosci..

[8] Causeret F., Coppola E., Pierani A. (2018). Cortical developmental death: Selected to survive or fated to die. Curr. Opin. Neurobiol..

[9] Ruijter J.M., Baker R.E., De Jong B.M., Romijn H.J. (1991). Chronic blockade of bioelectric activity in neonatal rat cortex grown in vitro: Morphological effects. Int. J. Dev. Neurosci..

[10] Heck N., Golbs A., Riedemann T., Sun J.J., Lessmann V., Luhmann H.J. (2008). Activity-dependent regulation of neuronal apoptosis in neonatal mouse cerebral cortex. Cereb. Cortex.

[11] Ikonomidou C., Bosch F., Miksa M., Bittigau P., Vöckler J., Dikranian K., Tenkova T.I., Stefovska V., Turski L., Olney J.W. (1999). Blockade of NMDA receptors and apoptotic neurodegeneration in the developing brain. Science.

[12] Fishbein I., Segal M. (2007). Miniature synaptic currents become neurotoxic to chronically silenced neurons. Cereb. Cortex.

[13] Golbs A., Nimmervoll B., Sun J.J., Sava I.E., Luhmann H.J. (2011). Control of programmed cell death by distinct electrical activity patterns. Cereb. Cortex.

[14] Ghosh A., Carnahan J., Greenberg M.E. (1994). Requirement for BDNF in activity-dependent survival of cortical neurons. Science.

[15] Allène C., Cattani A., Ackman J.B., Bonifazi P., Aniksztejn L., Ben-Ari Y., Cossart R. (2008). Sequential generation of two distinct synapse-driven network patterns in developing neocortex. J. Neurosci..

[16] Murase S., Owens D.F., McKay R.D. (2011). In the newborn hippocampus, neurotrophin-dependent survival requires spontaneous activity and integrin signaling. J. Neurosci..

[17] Wong F.K., Bercsenyi K., Sreenivasan V., Portalés A., Fernández-Otero M., Marín O. (2018). Pyramidal cell regulation of interneuron survival sculpts cortical networks. Nature.

[18] Blanquie O., Liebmann L., Hübner C.A., Luhmann H.J., Sinning A. (2016). NKCC1-mediated GABAergic signaling promotes postnatal cell death in neocortical Cajal-Retzius cells. Cereb. Cortex.

[19] Denaxa M., Neves G., Rabinowitz A., Kemlo S., Liodis P., Burrone J., Pachnis V. (2018). Modulation of apoptosis controls inhibitory interneuron number in the cortex. Cell Rep..

[20] Priya R., Paredes M.F., Karayannis T., Yusuf N., Liu X., Jaglin X., Graef I., Alvarez-Buylla A., Fishell G. (2018). Activity regulates cell death within cortical interneurons through a calcineurin-dependent mechanism. Cell Rep..

[21] Duan Z.R.S., Che A., Chu P., Modol L., Bollmann Y., Babij R., Fetcho R.N., Otsuka T., Fuccillo M.V., Liston C. (2020). GABAergic restriction of network dynamics regulates interneuron survival in the developing cortex. Neuron.

[22] Riva M., Genescu I., Habermacher C., Orduz D., Ledonne F., Rijli F.M., Lopez-Bendito G., Coppola E., Garel S., Angulo M.C. (2019). Activity-dependent death of transient Cajal-Retzius neurons is required for functional cortical wiring. eLife.

[23] Blanquie O., Yang J.W., Kilb W., Sharopov S., Sinning A., Luhmann H.J. (2017). Electrical activity controls area-specific expression of neuronal apoptosis in the mouse developing cerebral cortex. eLife.

[24] Nimmervoll B., White R., Yang J.W., An S., Henn C., Sun J.J., Luhmann H.J. (2013). LPS-induced microglial secretion of TNFα increases activity-dependent neuronal apoptosis in the neonatal cerebral cortex. Cereb. Cortex.

[25] Yang J.W., Hanganu-Opatz I.L., Sun J.J., Luhmann H.J. (2009). Three patterns of oscillatory activity differentially synchronize developing neocortical networks in vivo. J. Neurosci..

[26] Minlebaev M., Colonnese M., Tsintsadze T., Sirota A., Khazipov R. (2011). Early γ oscillations synchronize developing thalamus and cortex. Science.

[27] Molnár Z., Luhmann H.J., Kanold P.O. (2020). Transient cortical circuits match spontaneous and sensory-driven activity during development. Science.

[28] Hanganu I.L., Ben-Ari Y., Khazipov R. (2006). Retinal waves trigger spindle bursts in the neonatal rat visual cortex. J. Neurosci..

[29] Banfalvi G. (2017). Methods to detect apoptotic cell death. Apoptosis.

[30] Howe E.S., Clemente T.E., Bass H.W. (2012). Maize histone H2B-mCherry: A new fluorescent chromatin marker for somatic and meiotic chromosome research. DNA Cell Biol..

[31] Southwell D.G., Paredes M.F., Galvao R.P., Jones D.L., Froemke R.C., Sebe J.Y., Alfaro-Cervello C., Tang Y., Garcia-Verdugo J.M., Rubenstein J.L. (2012). Intrinsically determined cell death of developing cortical interneurons. Nature.

[32] Newman J.P., Fong M.F., Millard D.C., Whitmire C.J., Stanley G.B., Potter S.M. (2015). Optogenetic feedback control of neural activity. eLife.

[33] Wang Y., Song J.H., Denisova J.V., Park W.M., Fontes J.D., Belousov A.B. (2012). Neuronal gap junction coupling is regulated by glutamate and plays critical role in cell death during neuronal injury. J. Neurosci..

[34] Dupont E., Hanganu I.L., Kilb W., Hirsch S., Luhmann H.J. (2006). Rapid developmental switch in the mechanisms driving early cortical columnar networks. Nature.

[35] Sun J.J., Kilb W., Luhmann H.J. (2010). Self-organization of repetitive spike patterns in developing neuronal networks in vitro. Eur. J. Neurosci..

[36] Wagenaar D.A., Pine J., Potter S.M. (2006). An extremely rich repertoire of bursting patterns during the development of cortical cultures. BMC Neurosci..

[37] Weir K., Blanquie O., Kilb W., Luhmann H.J., Sinning A. (2015). Comparison of spike parameters from optically identified GABAergic and glutamatergic neurons in sparse cortical cultures. Front. Cell. Neurosci..

[38] Sheng M., Greenberg M.E. (1990). The regulation and function of c-fos and other immediate early genes in the nervous system. Neuron.

[39] Kowiański P., Lietzau G., Czuba E., Waśkow M., Steliga A., Moryś J. (2018). BDNF: A key factor with multipotent impact on brain signaling and synaptic plasticity. Cell. Mol. Neurobiol..

[40] Singh R., Letai A., Sarosiek K. (2019). Regulation of apoptosis in health and disease: The balancing act of BCL-2 family proteins. Nat. Rev. Mol. Cell Biol..

[41] Egorov A.V., Draguhn A. (2012). Development of coherent neuronal activity patterns in mammalian cortical networks: Common principles and local hetereogeneity. Mech. Dev..

[42] Khazipov R., Luhmann H.J. (2006). Early patterns of electrical activity in the developing cerebral cortex of humans and rodents. Trends Neurosci..

[43] Luhmann H.J., Sinning A., Yang J.W., Reyes-Puerta V., Stüttgen M.C., Kirischuk S., Kilb W. (2016). Spontaneous neuronal activity in developing neocortical networks: From single cells to large-scale interactions. Front. Neural Circuits.

[44] Kilb W., Kirischuk S., Luhmann H.J. (2013). Role of tonic GABAergic currents during pre- and early postnatal rodent development. Front. Neural Circuits.

[45] Luhmann H.J., Kirischuk S., Sinning A., Kilb W. (2014). Early GABAergic circuitry in the cerebral cortex. Curr. Opin. Neurobiol..

[46] Fong M.F., Newman J.P., Potter S.M., Wenner P. (2015). Upward synaptic scaling is dependent on neurotransmission rather than spiking. Nat. Commun..

[47] Turrigiano G.G., Leslie K.R., Desai N.S., Rutherford L.C., Nelson S.B. (1998). Activity-dependent scaling of quantal amplitude in neocortical neurons. Nature.

[48] Fenno L., Yizhar O., Deisseroth K. (2011). The development and application of optogenetics. Annu. Rev. Neurosci..

[49] Lignani G., Ferrea E., Difato F., Amarù J., Ferroni E., Lugarà E., Espinoza S., Gainetdinov R.R., Baldelli P., Benfenati F. (2013). Long-term optical stimulation of channelrhodopsin-expressing neurons to study network plasticity. Front. Mol. Neurosci..

[50] Movsesyan V.A., Stoica B.A., Faden A.I. (2004). MGLuR5 activation reduces beta-amyloid-induced cell death in primary neuronal cultures and attenuates translocation of cytochrome c and apoptosis-inducing factor. J. Neurochem..

[51] Gu X., Spitzer N.C. (1995). Distinct aspects of neuronal differentiation encoded by frequency of spontaneous Ca^2+^ transients. Nature.

[52] Balkowiec A., Katz D.M. (2002). Cellular mechanisms regulating activity-dependent release of native brain-derived neurotrophic factor from hippocampal neurons. J. Neurosci..

[53] Gundlfinger A., Breustedt J., Sullivan D., Schmitz D. (2010). Natural spike trains trigger short- and long-lasting dynamics at hippocampal mossy fiber synapses in rodents. PLoS ONE.

[54] Paulsen O., Sejnowski T.J. (2000). Natural patterns of activity and long-term synaptic plasticity. Curr. Opin. Neurobiol..

[55] Friedman L.K. (2006). Calcium: A role for neuroprotection and sustained adaptation. Mol. Interv..

[56] Flavell S.W., Greenberg M.E. (2008). Signaling mechanisms linking neuronal activity to gene expression and plasticity of the nervous system. Annu. Rev. Neurosci..

[57] Bito H., Deisseroth K., Tsien R.W. (1997). Ca2+-dependent regulation in neuronal gene expression. Curr. Opin. Neurobiol..

[58] Garaschuk O., Linn J., Eilers J., Konnerth A. (2000). Large-scale oscillatory calcium waves in the immature cortex. Nat. Neurosci..

[59] Kirmse K., Kummer M., Kovalchuk Y., Witte O.W., Garaschuk O., Holthoff K. (2015). GABA depolarizes immature neurons and inhibits network activity in the neonatal neocortex in vivo. Nat. Comm..

[60] Okuno H. (2011). Regulation and function of immediate-early genes in the brain: Beyond neuronal activity markers. Neurosci. Res..

[61] Park S., Koppes R.A., Froriep U.P., Jia X., Achyuta A.K., McLaughlin B.L., Anikeeva P. (2015). Optogenetic control of nerve growth. Sci. Rep..

[62] Tao X., West A.E., Chen W.G., Corfas G., Greenberg M.E. (2002). A calcium-responsive transcription factor, CaRF, that regulates neuronal activity-dependent expression of BDNF. Neuron.

[63] Brigadski T., Leßmann V. (2020). The physiology of regulated BDNF release. Cell Tissue Res..

[64] Chen S.D., Wu C.L., Hwang W.C., Yang D.I. (2017). More insight into BDNF against neurodegeneration: Anti-apoptosis, anti-oxidation, and suppression of autophagy. Int. J. Mol. Sci..

[65] Antonsson B., Conti F., Ciavatta A., Montessuit S., Lewis S., Martinou I., Bernasconi L., Bernard A., Mermod J.J., Mazzei G. (1997). Inhibition of bax channel-forming activity by Bcl-2. Science.

[66] Akhtar R.S., Ness J.M., Roth K.A. (2004). Bcl-2 family regulation of neuronal development and neurodegeneration. Biochim. Biophys. Acta.

[67] Kole A.J., Annis R.P., Deshmukh M. (2013). Mature neurons: Equipped for survival. Cell Death Dis..

[68] Ter Horst H.J., Sommer C., Bergman K.A., Fock J.M., van Weerden T.W., Bos A.F. (2004). Prognostic significance of amplitude-integrated EEG during the first 72 hours after birth in severely asphyxiated neonates. Pediatr. Res..

[69] Iyer K.K., Roberts J.A., Hellström-Westas L., Wikström S., Hansen Pupp I., Ley D., Vanhatalo S., Breakspear M. (2015). Cortical burst dynamics predict clinical outcome early in extremely preterm infants. Brain.

[70] Legéndy C.R., Salcman M. (1985). Bursts and recurrences of bursts in the spike trains of spontaneously active striate cortex neurons. J. Neurophysiol..

[71] Pfaffl M.W. (2001). A new mathematical model for relative quantification in real-time RT-PCR. Nucleic Acids Res..

